# Prevalence of Occult Hepatitis B Virus Infection in a Cohort of HIV-Positive Patients Resident in Sicily, Italy

**DOI:** 10.1155/2013/859583

**Published:** 2013-08-26

**Authors:** Fabio Tramuto, Carmelo Massimo Maida, Giuseppina M. E. Colomba, Paola Di Carlo, Francesco Vitale

**Affiliations:** ^1^Department of Sciences for the Health Promotion and Mother and Child Care “G. D'Alessandro”, Hygiene Section, University of Palermo, 133 Via del Vespro, 90127 Palermo, Italy; ^2^Department of Sciences for the Health Promotion and Mother and Child Care “G. D'Alessandro”, Infectious Diseases Section, University of Palermo, 133 Via del Vespro, 90127 Palermo, Italy

## Abstract

Occult hepatitis B virus (OBI) in HIV-infected groups is still debated, as well as the associated risk-factors and clinical significance. In this paper, we examined a total of 405 HBsAg-negative/HIV-infected patients enrolled from January 2007 to December 2009. Overall, the prevalence of OBI was 5.9% (95% confidence interval (CI_95%_): 3.8–8.7%); it was more frequently associated with “anti-HBc alone” serological marker (11.3%; adjusted odds ratio = 3.7, CI_95%_: 1.4–9.8), although it was also detected in the absence of any HBV serological marker (4.9%; CI_95%_: 2.3–9.1%). A low prevalence of anti-HCV-positive patients with OBI was found (3.1%; CI_95%_: 0.6–8.7%). HIV RNA plasma levels or other immunological/clinical characteristics were not significantly associated with OBI. All but one occult HBV infections were sustained by genotype D viral strains. OBI is relatively frequent in HIV-infected patients, although it does not seem to exert a relevant clinical impact. Viral genotypes in occult HBV infections reflect those circulating in the Mediterranean area.

## 1. Introduction

The term “occult” hepatitis B infection (OBI) is defined by the presence of HBV DNA in plasma and/or liver tissue of subjects who lack detectable hepatitis B surface antigen (HBsAg).

Occult HBV infections are more frequently detected in individuals with antibodies to hepatitis B core antigen (anti-HBc) [1], often as unique marker of HBV infection [2, 3]. Nevertheless, recent estimates suggest that up to 20% of individuals with occult HBV could be negative even for anti-HBc antibodies or any other serological indicator of exposure to HBV [4], indicating that diagnosis of occult HBV infection is still an unresolved issue [5].

The prevalence of occult HBV in HIV-positive individuals remains controversial, varying between 0% and 90% [6–10], and the clinical significance is still unclear.

Nevertheless, occult HBV infection has important implications in HIV coinfected groups, being associated with faster rate of liver disease progression toward cirrhosis and hepatocellular carcinoma [11].

Although the impact of occult HBV infection has been investigated in different countries [3, 7, 9, 12–14] and clinical settings [15], no data are available on the prevalence of OBI among HIV-positive individuals from our geographic area.

A cross-sectional retrospective study was carried out with the aim of assessing the prevalence, risk factors, and genotypic characteristics of occult HBV infection in a cohort of HIV-infected individuals living in Sicily, either naïve or treated with HAART.

## 2. Materials and Methods

### 2.1. Study Population

A cross-sectional retrospective study was carried out on a total of 405 HBsAg-negative/HIV-positive subjects (median age 41.0 years, 67.9% males) admitted to the Infectious Diseases ward of the University Hospital “P. Giaccone,” Palermo, Italy, from January 2007 to December 2009, in day-hospital regimen for clinical followup of HIV infection.

All demographic, clinical, and laboratory data were stored according to the Italian laws on privacy, and the research was conducted following the Helsinki Declaration statements. The study was reviewed and approved by the institutional review board of the university hospital.

### 2.2. Laboratory Analysis

Plasma samples of HIV-infected patients were collected and kept frozen at −80°C until further analysis. HBV/HCV serological markers of infection were evaluated for routinary examination by the use of third-generation enzyme-linked-immunosorbent assays (Vitros Immunodiagnostics, Ortho-Clinical Diagnostics, High Wycombe, UK), and anti-HCV-positive samples were confirmed with a third-generation recombinant-immunoblot assay (RIBA) (Ortho-Clinical Diagnostics, High Wycombe, UK).

In addition, for the baseline evaluation of liver injury, some biochemical parameters were tested: alanine/aspartate aminotransferase (ALT/AST), total/direct/indirect bilirubin, gamma-glutamyl-transferase, and alkaline phosphatase (Ortho-Clinical Diagnostics, High Wycombe, UK).

Detection of occult HBV infection was carried out in agreement with the key recommendations of EASL consensus conference [16]. In this regard, up to 1 mL of plasma sample collected from HBsAg-negative patients was used during RNA extraction, and three different in-house nested-PCR amplification assays were applied to detect pre-S/S, pre-Core/Core, and Pol HBV viral sequences, respectively [3]. Appropriate negative and positive controls were included in each PCR reaction.

In our study, a “case of occult HBV infection” was defined as a PCR-positivity in at least one of the three HBV domains defined, after a confirmation of its specificity by sequencing [3].

Viral nucleotide sequences obtained from each PCR-positive sample were submitted to the web-based genotyping program at the National Center for Biotechnology Information (NCBI) (http://www.ncbi.nlm.nih.gov/projects/genotyping/formpage.cgi) to identify the corresponding HBV genotype.

All tests were performed at the Molecular Epidemiology Laboratory of the Department of Sciences for Health Promotion and Mother and Child Care “G. D'Alessandro,” Hygiene section, University of Palermo, Italy.

### 2.3. Statistical Analysis

Descriptive statistics were calculated and reported for sociodemographic and clinical characteristics. Median and interquartile ranges (IQRs) were used for description of continuous variables, while frequency analyses for categorical variables were described with the use of percentages.

According to data distribution, comparisons of continuous variables were conducted by using Students *t*-test or Mann-Whitney *U* test, while categorical variables were compared with the Chi-square test or Fisher's exact test, as appropriate. A *P* value < 0.05 was considered to indicate statistical significance.

A logistic regression analysis was used to examine the association between occult HBV status and specific serological pattern of viral infection, and the results were expressed as odds ratios (ORs) with 95% confidence intervals (CIs).

Statistic analysis was performed by using STATA for Apple (version 12.1 MP, StataCorp, College Station, TX, USA).

## 3. Results

Table 1 describes the sociodemographic and serological characteristics of the study population. The study group consisted of 405 HBsAg-negative/HIV-positive subjects (275 males and 130 females, M : F ratio = 2.1) with a median age of 41.0 years (IQR = 14.2 years); females were slightly younger than males (40.3 years versus 41.7 years, resp.).

More than two-thirds of subjects (78.5%, *n* = 318/405) reported promiscuous sexual activity as risk behaviour associated to HIV positivity, while intravenous drug use (IVDU) was prevalent among HIV/HCV coinfected patients (44.9%, *n* = 44/98).

The cohort of subjects presented in this paper mainly consisted of native Italians (82.7%, *n* = 335/405), 15.6% (*n* = 63/405) were Africans, and only a minority originated from other geographic regions including Eastern Europe (1.7%, *n* = 7/405).

According to the HBV/HCV serological status, five different categories were identified. One hundred eighty-three HIV-positive individuals (45.2%) were negative to all of the serological markers of HBV infection performed in this study, 88 (21.7%) were anti-HBs + anti-HBc positive, 71 (17.5%) showed the serological profile “anti-HBc alone,” and 63 (15.6%) were positive to anti-HBs as unique marker of infection. Overall, 24.2% of HIV-positive subjects (*n* = 98/405) were coinfected with HCV; the anti-HBc alone pattern was more frequently detected in HCV c-infected individuals than in HCV-negative subjects (38.8% versus 10.7%; OR = 5.3, *P* < 0.001).

DNA sequences of hepatitis B viruses were detected in 24 out of 405 patients (Table 2(a)), corresponding to a cumulative prevalence of occult HBV of 5.9% (CI_95%_: 3.8–8.7%).

HBV DNA resulted more frequently associated to the serological pattern anti-HBc alone (11.3%, *n* = 8/71), followed by anti-HBs + anti-HBc (6.8%, *n* = 6/88), and no HBV markers (4.9%, *n* = 9/183). Moreover, HBV DNA sequences were detected in 3 out of 98 patients coinfected with HCV (3.1%), of whom two showed the pattern anti-HBc alone.

HIV RNA plasma levels were lower among HBV DNA-negative than -positive individuals (1.8 log_10_ HIV RNA copies/mL versus 2.8 log_10_ HIV RNA copies/mL), although about half of OBI cases (41.7%, *n* = 10/24) had undetectable HIV viral load (≤50 HIV RNA copies/mL).

Overall, one hundred forty-five subjects (35.8%) were naïve to HAART. The most part of patients with known HAART regimens (75.1%, *n* = 160/213) were taking a therapy including lamivudine, with similar proportions between HBV DNA-negative and -positive subjects (74.5%, *n* = 149/200 versus 84.6%, *n* = 11/13). The median time of exposure to antiretrovirals did not differ between OBI and non-OBI patients.

Immune status was also explored comparing the two HBV DNA groups. The CD_4_^+^ cell counts of HIV-infected patients were found to be similar between groups (388 cells/mL versus 419 cells/mL, *P* = 0.758), and a higher proportion of subjects with CD_4_^+^ counts ≤ 200 cells/mL was observed in the HBV-DNA negative group in respect to the HBV DNA-positive group (22.9%, *n* = 75/328 versus 20.0%, *n* = 4/20), although the difference was not statistically supported.

Plasma concentrations of biochemical indicators of liver damage were substantially similar in the two HBV groups considered, either in the cohort as a whole or in HAART-naïve subjects (data not shown).

Finally, each HIV-positive patient with occult HBV infection was genetically characterized for viral HBV genotype classification. Almost the totality of patients with OBI were collected during the period 2008-2009 and were infected by genotype D viruses; only one subject, native of sub-Saharan Africa, harboured a genotype E hepatitis B virus (Table 2(b)).

## 4. Discussion

In the present study, a cohort of 405 HBsAg-negative/HIV-infected individuals was investigated in order to assess the impact of occult HBV infection in Sicily, and an overall prevalence of 5.9% (CI_95%_: 3.8–8.7%) was found.

In Italy, the prevalence of OBI has been previously evaluated in different clinical settings [3, 9, 14, 17–19], and, to our knowledge, this is the first work carried out in our geographic area on a cohort of HIV-infected patients.

In this specific high-risk group, several studies have been conducted worldwide and the available data on the frequency of occult HBV infection are widely divergent, ranging from 0% to more than 90% [6–10], mostly depending on differences in terms of sensitivity limit of the assay used (standard/nested PCR, real-time PCR, etc.), number of HBV DNA domains examined, biological compartment explored (liver, plasma, or both), and composition of the study populations [1, 20, 21].

This work confirms that occult HBV can be detected either in patients with serological evidence of past “apparently resolved” HBV infection, but also in individuals with no evident history of exposure to HBV [22].

The prevalence of OBI presented in this paper is quite similar to that reported in European countries such as The Netherlands [6], consistently higher than that observed in Spain [13], France [21], or Taiwan [23] (0.7%, 0.6%, and 2.3%, resp.), but lower than that reported in countries either at low or high rates of chronic HBV [7, 20, 24].

In Sicily, the impact of OBI has been recently investigated in different groups of subjects belonging to low- and high-risk HBV exposure, such as general population, intravenous drug users, patients with hepatocellular carcinoma, and immigrants from geographic areas with high rate of HBV infection [3, 14].

In accordance to other authors [9, 22, 25, 26], our findings add consistency to the role of the anti-HBc alone profile as the most adequate serological surrogate of OBI, being the only factor significantly associated to a greater probability of OBI detection, although higher prevalence of anti-HBc alone does not necessarily reflect significantly higher frequency of OBI [3, 9, 14, 22, 25, 26]. HIV infection has been proposed to have a major effect on OBI, leading to more consistent levels in symptomatic HIV as compared to asymptomatic HIV [27], being significantly associated with lower CD_4_^+^ cell counts [6].

In our experience, although HBV DNA-positive patients showed higher levels of HIV RNA, the median CD_4_^+^ cell counts were not significantly different when compared to HBV DNA-negative group, and OBI cases were quite similarly represented independently to CD_4_^+^ plasma levels.

Overall, the contribution of occult HBV to liver damage remains unclear. Although ALT/AST flares have been observed in HCV-positive patients in association with occult HBV [28], in our series we did not find abnormal levels of biochemical indicators of liver injury in OBI-positive patients, in accordance to the overall trend in the literature [9, 18, 29], also in correlation to the immunosuppressed state of HIV-positive subjects [12, 30].

In general, HCV coinfection has been considered as one of the main reasons for inducing OBI [31], and prevalences of occult HBV infection have been reported to be significantly higher in HCV chronically coinfected patients as compared to HCV-negative individuals [32, 33].

In Italy, the impact of OBI in cohorts of HIV-positive patients coinfected with hepatitis C virus was recently investigated, either in plasma samples or liver biopsies [9, 18], reporting consistent prevalences of OBI and suggesting a strong correlation with HCV. Nevertheless, in accordance with other recently published studies [34, 35], in our cohort occult HBV infection was uncommon among anti-HCV positive subjects, and the high frequency of HAART-treated individuals within this specific group (most of them assuming a lamivudine-based therapy) could partially explain the low prevalence found [36, 37].

The positive association between HIV and chronic hepatitis B infection is well known, in terms of higher levels of HBV DNA and detection of HBV antigens [38, 39], especially before the availability of HAART. More recently, the widespread introduction of HAART in clinical practice, commonly associated with HIV/HBV dually active drugs (i.e. lamivudine, tenofovir, and emtricitabine), plays an important role in the suppression of virus replication [17], leading to lower levels of OBI in HAART-treated HIV-positive subjects.

However, as similarly reported by other authors in Italy [9, 40], in our study population occult HBV infections were found either in HAART naïve or treated patients, and lamivudine-based HAART did not exert an important effect on HBV DNA detectability.

Finally, in this study, the most part of occult infections were sustained by genotype D HBV viral strains, which is consistent with the genotypes circulating in Sicily [14]. In this regard, although it has been suggested that in occult HBV infection preferentially occurring genotype D viruses [9, 35, 41–43], HBV genotypes other than D, including the C genotype [44, 45] as well as A, G, and E genotypes [14, 22, 46], have been detected in OBI.

Our findings should be interpreted in light of the possible limitations of this study, including the cross-sectional retrospective design, the use of a single time point testing without any followup, and the biological compartment explored to detect HBV-DNA. Moreover, the limited sample size may have reduced our ability to detect differences between groups, and the results, as such, may not be generalizable to other settings.

Nevertheless, despite these limitations, our results add evidence to the knowledge in this field, in a geographic area where the increasing trend in migration flows could play an important role in promoting a modification of the local HBV epidemiology.

## 5. Conclusions

The surveillance of occult HBV infection and its genetic variability is recommended to better evaluate the viral dynamics and their role in the outcome of the liver damage in HIV/HCV coinfected as well as in healthy individuals with HBV serological pattern suggestive of latent HBV infection.

## Figures and Tables

**Table 1:** Socio-demographic and serological characteristics of 405 HBsAg-negative/HIV-infected individuals.

Characteristic (*n* (%), by column)		No HBV markers	Anti-HBs + Anti-HBc	Anti-HBc alone	Anti-HBs alone	Anti-HCV
Study population (% by row)	**405**	**183 (45.2)**	**88 (21.7)**	**71 **(17.5)*	**63 (15.6)**	**98 **(24.2)^∆^
Sex						
Male	275 (67.9)	125 (68.5)	59 (66.7)	50 (70.4)	41 (64.1)	75 (76.5)
Female	130 (32.1)	58 (31.5)	29 (33.3)	21 (29.6)	22 (35.9)	23 (23.5)
M : F ratio	2.1	2.1	2.0	2.4	1.9	3.3
Age (years, median (IQR))	**41.0 (14.2)**	**41.0 (12.5)**	**43.9 (14.4)**	**43.8 (11.2)**	**32.0 (20.2)**	**45.1 (5.8)**
≤20 years	15 (3.7)	6 (3.3)	3 (3.4)	2 (2.8)	4 (6.3)	1 (1.0)
21–30 years	63 (15.5)	23 (12.6)	8 (9.1)	7 (9.9)	25 (39.7)	3 (3.1)
31–40 years	108 (26.7)	57 (31.1)	24 (27.3)	15 (21.1)	12 (19.0)	13 (13.3)
≥41 years	219 (54.1)	97 (53.0)	53 (60.2)	47 (66.2)	22 (34.9)	81 (82.7)
Mode of HIV transmission						
Heterosexual	197 (48.6)	92 (50.3)	34 (38.6)	40 (56.3)	31 (49.2)	35 (35.7)
Homosexual	121 (29.9)	63 (34.4)	24 (27.3)	9 (12.7)	25 (39.7)	15 (15.3)
IVDU	71 (17.5)	21 (11.5)	23 (26.1)	21 (29.6)	6 (9.5)	44 (44.9)
Other/unknown	16 (4.0)	7 (3.8)	7 (8.0)	1 (1.4)	1 (1.6)	4 (4.1)
Geographic origin						
Italy	335 (82.7)	160 (87.4)	67 (76.1)	51 (71.8)	57 (90.5)	91 (92.9)
Africa	63 (15.6)	20 (10.9)	17 (19.3)	20 (28.2)	6 (9.5)	7 (7.1)
Eastern Europe	4 (1.0)	2 (1.1)	2 (2.3)	0 (0)	0 (0)	0 (0)
Other	3 (0.7)	1 (0.5)	2 (2.3)	0 (0)	0 (0)	0 (0)

*Anti-HCV positive versus anti-HCV negative = 38.8% versus 10.7% (OR = 5.3, CI_95%_: 3.0–9.3, *P* < 0.001).

^∆^ *n* = 29/405 (7.2%): HIV/HCV coinfected subjects negative to all of the serological markers of HBV infection.

IVDU: Intravenous drug use.

**Table:** (a)

Characteristic (*n* (%), by column)	Total	HBV DNA negative	HBV DNA positive	CI_95%_ or *P* value
Study population (*n* (%), by row)	**405**	381 (94.1)	24 (5.9)	3.8–8.7%
Serological markers of infection^□^				
No HBV markers	183 (45.2)	174 (95.1)	9 (4.9)	2.3–9.1%
Anti-HBs + anti-HBc	88 (21.7)	82 (93.2)	6 (6.8)	2.5–14.2%
Anti-HBc alone	71 (17.5)	63 (88.7)	8 (11.3)^∗,∆^	5.0–21.0%
Anti-HBs alone	63 (15.6)	62 (98.4)	1 (1.6)	0.1–8.5%
Anti-HCV	98 (24.2)	95 (96.9)	3 (3.1)	0.6–8.7%
HIV viral load				
HIV RNA [log_10_ copies/mL, median (IQR))	1.8 (3.0)	1.8 (3.0)	2.8 (3.1)	0.834
HIV RNA (≤50 copies/mL)	196 (47.9)	186 (94.9)	10 (5.1)	0.775
HAART regimen				
Naïve	145 (35.8)	138 (95.2)	7 (4.8)	
HAART including lamivudine	160 (39.5)	149 (93.1)	11 (6.9)	
HAART without lamivudine	53 (13.1)	51 (96.2)	2 (3.8)	
HAART not specified	47 (11.6)	43 (91.5)	4 (8.5)	
Months of treatment (median (IQR))	55.2 (53.9)	54.9 (55.1)	58.1 (31.2)	0.954
Immunological parameters (*n* = 348)				
CD_4_^+^ cell counts (median (IQR))	390 (386)	388 (382)	419 (368)	0.758
≤200 cells/mL	79 (22.7)	75 (94.9)	4 (5.1)	
201–499 cells/mL	143 (41.1)	136 (95.1)	7 (4.9)	
≥500 cells/mL	126 (36.2)	117 (92.9)	9 (7.1)	

**Table:** (b)

Case no.	Sex	Age (years)	Geographic origin	Year of sampling	Risk factor	Anti-HBs	Anti-HBc	Anti-HCV	Genotype
1	F	22.3	Eastern Europe	2007	Hetero	−	−	−	D
2	F	26.8	Sub-Saharan Africa	2008	Hetero	−	+	−	D
3	F	36.2	Sub-Saharan Africa	2009	Hetero	−	−	−	D
4	M	41.0	Sub-Saharan Africa	2009	Hetero	−	+	−	E
5	M	40.0	Italy	2008	Hetero	−	+	−	D
6	M	51.8	Italy	2008	Hetero	+	+	−	D
7	F	42.6	Italy	2008	Hetero	−	−	−	D
8	F	30.3	Italy	2008	Hetero	−	−	−	D
9	M	43.9	Italy	2008	Hetero	−	−	+	D
10	M	34.4	Italy	2008	IVDU	+	+	−	D
11	F	38.0	Italy	2008	Hetero	−	+	−	D
12	M	47.8	Italy	2008	Omo	−	−	−	D
13	M	53.1	Italy	2009	IVDU	+	+	−	D
14	M	60.0	Italy	2009	Hetero	−	+	−	D
15	M	44.7	Italy	2009	Hetero	+	+	−	D
16	F	39.8	Italy	2009	Hetero	+	−	−	D
17	F	38.9	Italy	2009	Hetero	−	+	−	D
18	F	47.6	Italy	2009	Hetero	−	−	−	D
19	M	40.3	Italy	2009	Omo	−	−	−	D
20	M	43.2	Italy	2009	IVDU	−	+	+	D
21	F	39.4	Italy	2009	Hetero	+	+	−	D
22	M	35.2	Italy	2009	Omo	+	+	−	D
23	M	46.9	Italy	2009	Hetero	−	+	+	D
24	M	29.7	Italy	2009	Omo	−	−	−	D

^□^Frequency and pattern of HBV serological markers are not mutually exclusive.

*OR = 3.7 (1.4–9.8), Mantel-Haenszel OR adjusted for anti-HCV positivity.

^∆^OR = 4.0 (1.5–10.7), Mantel-Haenszel OR adjusted for anti-HCV positivity. Analysis restricted to 145 HAART-naïve patients.

CI_95%_; 95% confidence interval; OR: odds ratio; IQR: interquartile range; Hetero: heterosexual; Omo: homosexual; IVDU: intravenous drug use.
